# Drivers of Bird Species Richness within Moist High-Altitude Grasslands in Eastern South Africa

**DOI:** 10.1371/journal.pone.0162609

**Published:** 2016-10-05

**Authors:** David H. Maphisa, Hanneline Smit-Robinson, Les G. Underhill, Res Altwegg

**Affiliations:** 1 South African National Biodiversity Institute, Private Bag X7, Claremont 7735, South Africa; 2 Statistics in Ecology, Environment and Conservation, Department of Statistical Sciences University of Cape Town, Rondebosch 7701, South Africa; 3 Animal Demography Unit, Department of Biological Sciences, University of Cape Town, Rondebosch 7701, South Africa; 4 BirdLife South Africa, Private Bag X5000 Parklands, 2121, South Africa; 5 Applied Behavioural Ecological & Ecosystem Research Unit (ABEERU), UNISA, Private Bag X6, Florida, 1717, South Africa; 6 African Climate and Development Initiative, University of Cape Town, Rondebosch 7701, South Africa; University of Sydney, AUSTRALIA

## Abstract

Moist high-altitude grasslands in South Africa are renowned for high avifaunal diversity and are priority areas for conservation. Conservation management of these areas conflicts with management for other uses, such as intensive livestock agriculture, which requires annual burning and leads to heavy grazing. Recently the area has become target for water storage schemes and renewable electricity energy projects. There is therefore an urgent need to investigate environmental factors and habitat factors that affect bird species richness in order to optimise management of those areas set aside for conservation. A particularly good opportunity to study these issues arose at Ingula in the eastern South African high-altitude grasslands. An area that had been subject to intense grazing was bought by the national power utility that constructed a pumped storage scheme on part of the land and set aside the rest for bird conservation. Since the new management took over in 2005 the area has been mostly annually burned with relatively little grazing. The new management seeks scientific advice on how to maintain avian species richness of the study area. We collected bird occurrence and vegetation data along random transects between 2006 and 2010 to monitor the impact of the new management, and to study the effect of the habitat changes on bird species richness. To achieve these, we convert bird transect data to presence only data to investigate how bird species richness were related to key transect vegetation attributes under this new grassland management. First we used generalised linear mixed models, to examine changes in vegetation grass height and cover and between burned and unburned habitats. Secondly, we examined how total bird species richness varied across seasons and years. And finally we investigated which habitat vegetation attributes were correlated with species richness of a group of grassland depended bird species only. Transects that were burned showed a larger decrease in vegetation cover compared to transects that were not burned. Grass height increased over time. Bird species richness was highest in summer compared to other seasons and increased over time. Overall bird species richness increased over the three summer surveys but species richness of birds that prefer heavily grazed habitat showed little change over the three years. Changes in bird species richness were best explained by the model with grass height for combined species richness of grassland depended birds but also for birds that prefer heavy grazing when treated alone. On one hand birds that prefer moderate grazing were best explained by a null model. However, overall bird species richness was better positively correlated to grass height than grass cover or dead grass. We conclude that frequent burning alone with relatively reduced grazing led to higher but less dense grass, which benefited some species and disadvantaged others. We suggest that management of this grassland use combination of fire and grazing and leave some areas unburned to accommodates birds of various habitat needs.

## Introduction

Worldwide, grasslands have been destroyed and are in need of urgent protection [1–3]. In South Africa, loss of habitat in the eastern, moist, high-altitude grasslands is of concern because the area harbours large avian diversity and endemism, including birds that are both nationally and globally threatened [4]. Only about 2% of grassland in the area is formally protected with the rest of the area under private ownership characterized by annual burning and heavy grazing[5–8]. Threats affecting the grassland birds in the area include land transformation due to agricultural activities and human settlement [9] but also inappropriate use of fire and grazing [5,10–12]. New, more worrisome developments within upland grassland of southern Africa includes the construction of water storage reservoirs and pumped storage schemes to generate electricity to meet increasing human[13,14]. Because high-altitude areas are exposed and are characterized by high wind speeds [15], they are also potential sites for wind farms. Globally, production of electricity from wind energy is expected to increase as an alternative to coal [16]. An additional threat comes from the transmission lines that convey electricity to the national grid. In southern Africa, upland grasslands are a stronghold for large threatened birds [17,18] and these new forms of energy development are likely to negatively impact on such birds[19]. A study from Scotland and northern England found that ground nesting birds are also threatened by above-ground electrical infrastructure [16]. With the current levels of habitat loss and threats from new energy developments, conservation of birds (and also other biota) must focus on identifying and managing key habitat vegetation attributes that sustain bird population(s) [20,21].

In this study we investigate environmental and habitat variables that drives avian species richness in the area. To do so we use data collected within the boundaries of Eskom Ingula Pumped Storage Scheme boundaries between 2005/06–2010/11. The Scheme is a prime example of the difficult balance between human land use and conservation. When Eskom was given permission to build a pumped storage scheme in a sensitive environment area, it was required to buy additional land with the aim of mitigating the loss of biodiversity during the construction of the scheme and to maximise biodiversity conservation beyond the construction of the scheme [14].

Ingula and surrounding high-altitude grasslands has a rich avifauna some of which are regionally threatened endemics while others are globally threatened and include summer Palearctic migrants [14]. Understanding bird-habitat interaction is central to implementing appropriate management tools to retain avifaunal diversity in the face of development in the area.

Before 2005, the Ingula property too was privately owned and used mainly for commercial cattle farming with fire used annually to optimise cattle feed. Impact assessment studies carried out prior to construction of the pumped storage scheme recommended that cattle be removed and replaced by game, because cattle were responsible for degradation of the wetland and surrounding grasslands [22]. Mentis [22] further recommended that the area be block-burned every other year. Cattle belonging to the commercial farmers were removed during late 2005. However, a relatively small herd of cattle belonging to previous commercial farmers’ tenants remained on site to be resettled by Eskom at some later stage. As of 2013, although the study site was blocked burned every fire season leaving some blocks unburned as per recommendations of [22] annual burning persisted on site and was largely caused by tenants [14].

With the current increase in threats facing the moist, high-altitude grasslands [8,9,15] and in order to prioritise limited conservation resources to conduct effective conservation planning, a better understanding of avian species richness patterns and habitat suitability is needed [23]. In this study we specifically, investigate how grassland habitat and bird species richness responded to these new grassland management policies. Current grassland management needs scientific advice on how to maintain and monitor avian species richness of the study area. Species richness is a metric of many monitoring projects [24]. Grass height and cover are the key vegetation attributes associated with bird habitat selection [20] or bird species richness. We perceived habitat mosaic defined in terms of range of grass heights and covers brought about by fire and grazing as key drivers of bird habitat suitability [25,26]. First we used transect vegetation data to investigate how grass cover and height varied between the summers of 2006/07, 2007/08 and 2010/11 and investigated which factors possibly drove these changes. Next we tested how total birds species richness changed across four seasons. Secondly, using the summer data only we investigate how bird species richness of birds that depend on grassland to feed and breed changed over the three years and we tested which vegetation attributes explained the observed change. Third we split depend grassland birds into birds that prefer heavy grazing and those that prefer moderate grazing. For each group we tested how species richness changed over the three years and likewise investigated how grazing and burning explained the observed changes. And finally, we tested how total bird species richness of this birds are correlated with grass height, cover and amount of dead grass across the transects. Based on these study outcomes we make recommendations on how to manage these grasslands to maintain and monitor avian species richness into the future.

## Materials and Methods

### Ethical statement

The field study was designed by the main author and fieldwork was carried out by the main author too while employed as a site ornithologist to advice the Ingula Partnership (Eskom and BirdLife South Africa) on how to maintain species richness of the area through appropriate grassland management. We further confirm that the funders (Ingula Partnership/Mazda Wildlife) had no role in study design, data collection and analysis, decision to publish, or preparation of the manuscript. We also confirm that our study did not involve the capture of live specimens or protected species and thus requiring no further applications/permits from the national/provincial bodies. The study area was confined to the land owned by Ingula Partnership (first author employer) and thus requiring no further permission/permits to carry out the study.

### Description of study site

Ingula is situated *c*. 23km north-east (28°14' S, 29°35' E) of the village of Van Reenen at altitudes of 1200 to 1700m asl and covers *c*. 8 000ha. It straddles the escarpment and two provinces: KwaZulu-Natal and Free State. The average altitude below the escarpment is 1200m asl and 1700m asl above the escarpment. The latter part is dominated by sweet and sour grassland vegetation type [27], characterised by the grass *Themeda triandra* and is used to support commercial livestock in summer. The area below the escarpment is dominated by *Hyparrhenia-Cybompogon* grasses and has been modified into fields and alien plantations and therefore is considered of less conservation priority compared to the upper site [14].

### Bird sampling

Using 1:50 000 topographic maps, 35 random transects of length 500m were placed perpendicular to farm vehicle tracks every 2 km [5] across the 8 000ha of Ingula, avoiding locations that were too rocky or too steep. We surveyed birds to within 150m on both sides of each transect line, once per season (winter: May-July, spring: August-October, summer: November-January, autumn: February-April) between the summers of 2006/07, 2008/09 and 2010/11. Weather permitting we sampled both birds and vegetation within a relatively short time apart on the each transect. Since some of the transects were lost due to construction, we only sampled seventeen of the 35 original transects during the summer of 2010/11. The fixed-width strip transects method involves counting and identifying all bird species seen or heard within a pre-determined distance of the line travelled [28]. This is widely used method to estimate bird abundance, species habitat preferences and species richness for monitoring conservation programmes [29]. For this analysis, we use the number of species recorded per transect rather than density as a measure of species richness[29]. The survey was carried out by one individuals mostly during early morning (07h00 – 11h00) but extended into mid-afternoon (15h00 – 16h00), when birds are most active [5]. The survey was only extended to mid-afternoon when survey time was limiting given the unpredictable weather conditions of the study area and the large area under survey. No surveys were carried out under wet conditions or when visibility was poor.

### Measurement of vegetation and land cover variables

Vegetation at each transect was surveyed in summer only after each bird survey for the three years; 2006/07, 2007/08 and 2010/11. We used a light metal steel quadrat to sample vegetation [5]. The quadrat consisting of 30cm x 30cm, nine equal squares, was thrown randomly, twice in every 100m along each 500m transects where a bird survey had been conducted earlier as close to bird survey as possible. In each quadrat, we recorded number of grid squares out of nine with grass, bare soil; forbs or stones. In addition, grass height (cm) was recorded at four corners of the frame. Our main interest is how grass cover and height affect bird species richness. We also recorded intensity of grazing along each transect according to light, medium or heavy, based on visual evidence of grass clipping or habitat trampling by animals. During all summer surveys, we recorded additional information on when each transect was last burned or not (eg. early or late spring, early summer or late summer). However, because of arson leading to several fires each year for the duration of our study, it was not easy to keep track of exactly when each transect was burned and therefore this information was reduced to burned or not burned. The topography around each transect was categorised into four types (plateau top, shallow slope, steep slope or valley bottom).

### Data analysis and statistical modeling

All data analyses were carried out in R [30] using generalised linear mixed effects models (GLMMs) [31,32] through function lmer in package lme4 v 1.1–7 [33]. We treated transect as random effect in all analyses to account for the repeated-measures nature of the data. Each analysis involved model selection using the Akaike Information Criterion (AIC) to rank the models where model with the lowest AIC was chosen as the best fitting model[34–37]. All candidate model sets included a model with no covariates (constant) with transect only as a random effect [38,39].

We started by comparing changes in grass cover and height over the three summers (2006/07, 2007/08 and 2010/11). The main reason for the removal of livestock belonging to commercial farmers was to encourage habitat to recover from many years of heavy grazing. For this we treated grass cover and grass height as response variables and year as fixed effect. We treated year as fixed effect because we expected habitat and bird species richness to vary amongst years [40]. Based on a prior hypothesis about variables that could potentially explain changes in grass cover or height we considered seven competing models. Because grass cover was a proportion (number of grids covered by grass out of nine) we assumed a binomial distribution and logit link function [41]. Following from the same procedure as above we compared the effect of fire (burned or not burned) on transect grass cover. For grass height, we assumed normally distributed errors and used the identity link function [42]. To reduce heteroscedasticity of the residuals, we log-transformed grass height before analysis [43]. We added a model with no variables where species richness was determined only by transect random effects as the ninth model.

First we tested how total bird species richness of all birds seen within 150m range changed over the four season and for this we treated a transect as a random effect and season as a fixed effect. But our primary interest was on how grass height and cover affected bird species richness because this two attributes can be managed with burning and grazing. Maphisa et al. [5] found out that sometimes threatened grassland birds co-exist demanding conflicting grassland management recommendations. We therefore divided bird data into birds that prefers heavy grazing and birds that are associated with moderate grazing. Next we merged vegetation data and birds data in r [30] and tested how bird species richness changed over the three years and investigated which transect habitat variables best explained the changes. For each group we tested how species richness changed in three years and investigated which transects vegetation attributes explained the changes. Because we converted distance sampling data into presence only data [29], we define species richness as a number of species seen per transect. Therefore our (GLMM) for species richness assumed a Poisson distribution of the data with a log link function [44]. We considered seven habitat candidate models bearing in mind that other transects attributes could also affect species richness [14]. We used our previous field knowledge [5] plus our field observations to choose candidate models. All our models included a null model (model with no covariates) with transect only as a random effect. In a final analysis we tested how species richness was correlated individually to grass cover, grass height, and proportion of ground covered by dead grass because these variables are important habitat attributes for grassland birds [5,20].

## Results

### Changes in grass cover and height during the survey period

**Fig 1:**
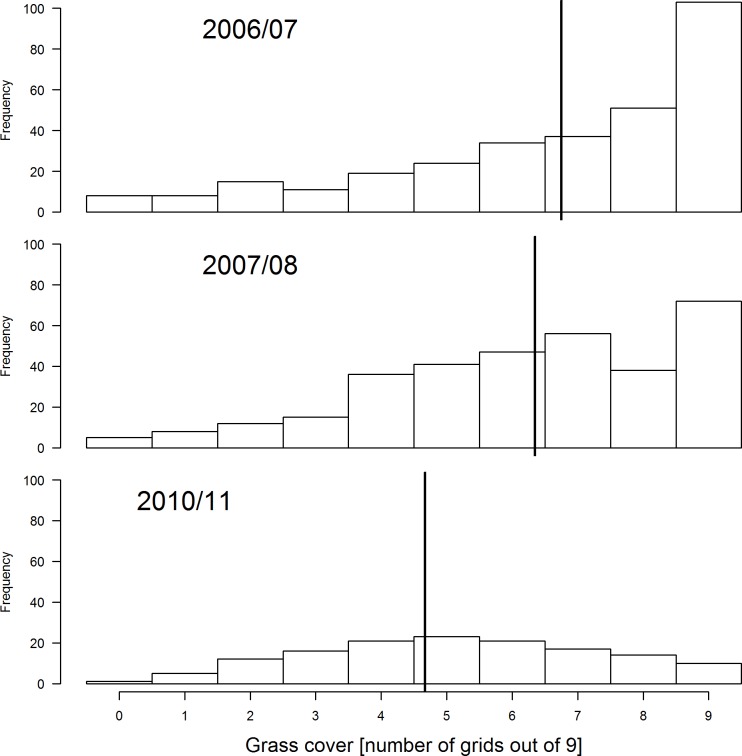
Comparisons of grass cover at Ingula over the three years of surveying. Year is treated as fixed effect and transect as a random effect. The vertical black solid line represents the mean according to the best model (Table 1) and the histograms show the distribution of the raw data. The data consisted of the count out of nine squares in each sampling grid that fell on grass.

**Fig 2:**
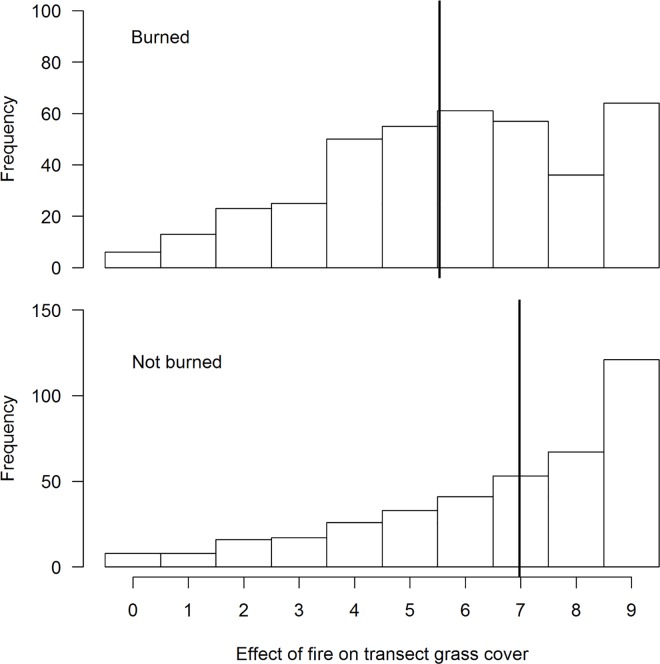
Comparison of influence of fire on grass cover along transects that were burned and those that were not burned at Ingula using summer data for three years (2006/7, 2007/08 and 2010/11). Year is a treated as a fixed effect and transect as a random effect. **Bold** vertical lines show the estimated means according to the best model (Table 1).

**Fig 3:**
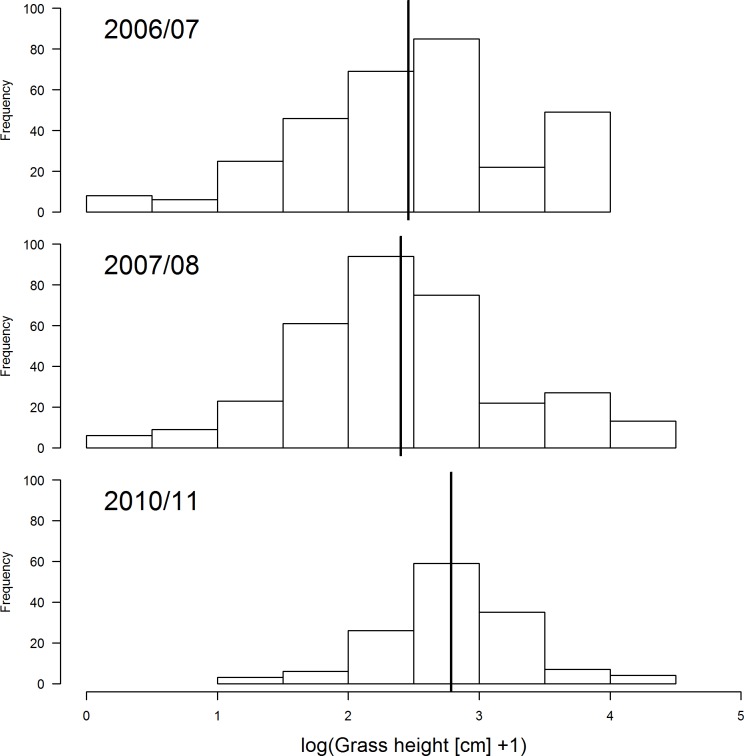
Comparison of grass height during the three summers of survey at Ingula (2006/07, 2007/08 and 2010/11). Year is treated as a fixed effect and transect as a random effect. **Bold** vertical lines represent the estimated means according to the best model (Table 2).

There was a decrease in the amount of grass cover during the three years (2007/07, 2007/08 and 2010/11) (Fig 1). Transects that had not been burned had denser cover compared to those that were burned (Fig 2). This change in grass cover was best explained by the model that combined years and fire by a wide margin compared to other competing models (Table 1). On the other hand, average grass height was slightly higher during 2010/11 compared to the other two summer surveys (Fig 3). A combination of fire and year best explained variation in average grass height better than other candidate models (Table 2).

**Table 1:** Generalized linear mixed models describing the effect of years, fire, grazing or topography on grass cover (the number of grid squares that were covered by grass, out of nine). Fire had two levels (burned vs not burned), grazing had three levels (not grazed, lightly grazed and heavily grazed) and topography had four levels. The models assume a binomial response and logit link function. Transect was treated as a random effect in all models such that a constant model has transect as random effect only. K is the number of parameters in each model including the intercept, Delta AIC is the difference between each model and the model with the lowest AIC. The model with the lowest AIC is the best model.

Models	K	Log likelihood	Delta AIC	Akaike weight
Constant	2	-2005.472	274.985	1.94E-60
Year	4	-1924.597	117.236	3.49E-26
Fire	3	-1928.588	123.219	1.75E-27
**Year + fire**	**5**	**-1864.979**	**0**	**1**
Grazing	4	-1996.930	261.903	1.34E-57
Topography	5	-2004.307	278.656	3.10E-61

**Table 2:** Linear mixed effects models for the effects of year, fire (two levels), grazing (3 levels) and topography (4 levels) on grass height across the three surveys. The models assumed a normally distributed response with grass height log-transformed. Transect was treated as a random effect in all models and therefore the constant model included transect as a random effect. K is the number of parameters in each model including the intercept and residual variance, Delta AIC is the difference between each model and the model with the lowest AIC. The model with the lowest AIC is the best model.

Models	K	Log likelihood	Delta AIC	Akaiki weight
Constant	3	-913.67	179.38	1.10E-39
Year	5	-902.17	160.38	1.50E-35
Fire	4	-1928.59	155.69	1.60E-34
**Year + fire**	**6**	**-820.98**	**0**	**1**
Grazing	5	-857.67	71.377	3.20E-16
Topography	6	-911.59	181.228	4.40E-40

### Bird species richness across seasons, years and habitat influence

In total, 76 species (S1 Appendix) were recorded across the 35 transects during three summers, two winters, two autumns and one spring. The list includes species that are not necessarily grasslands birds but were seen feeding in the vicinity of transects during the survey. Out of 76 species, 10 were classified as nationally threatened according to Taylor et al. [45]. The endemic and threatened Yellow-breasted Pipit *Anthus chloris* was the 12^th^ most common and widespread species recorded in all seasons in 16 out of 35 transects surveyed.

**Fig 4:**
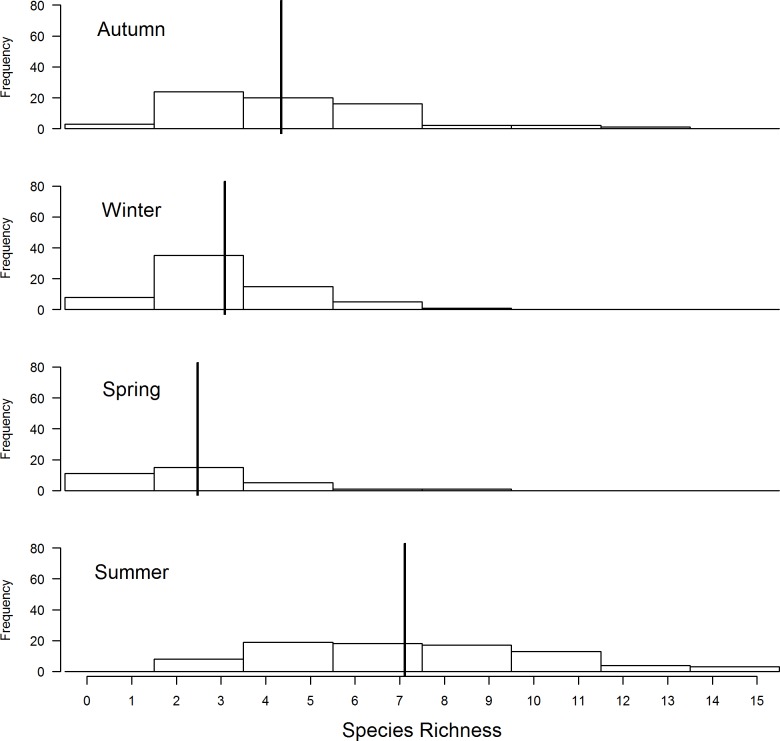
Comparison of Ingula bird species richness of all birds seen within 150m across the four seasons using transect data collected between 2006/07 to 2010/11. The data come from three summers, two autumns, two winters and one spring survey and only half the total number of transects were surveyed during summer 2010/11. Vertical lines show the estimated means obtained from a generalized linear mixed effects model that treated season as a fixed effect and transect as a random effect.

**Fig 5:**
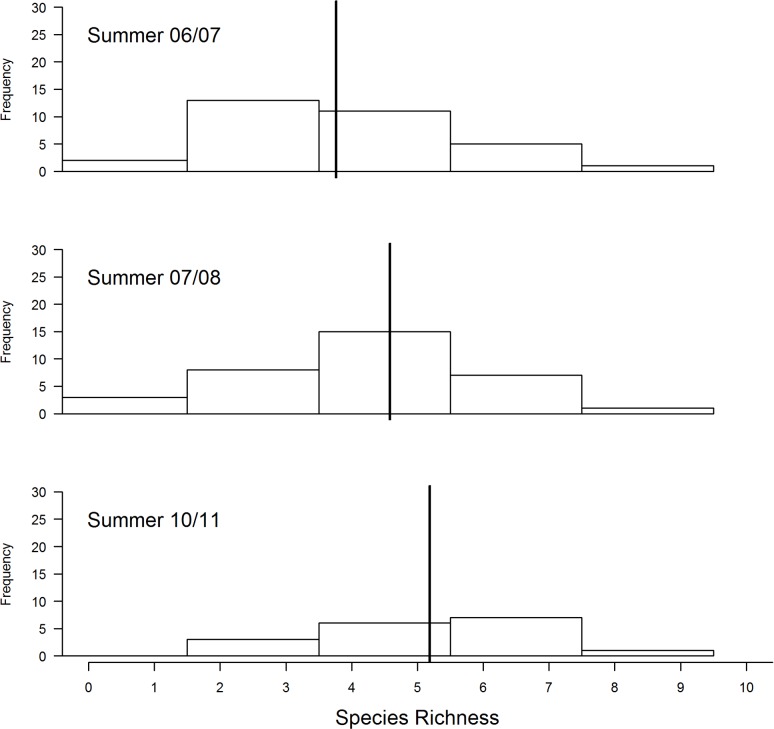
Comparison of total bird species richness (combining birds that prefer heavy grazing with birds that prefer light grazing) at Ingula using data from summer surveys only. Year is treated as a fixed effect and transect as a random effect. **Bold** bars represent the estimated means.

**Fig 6:**
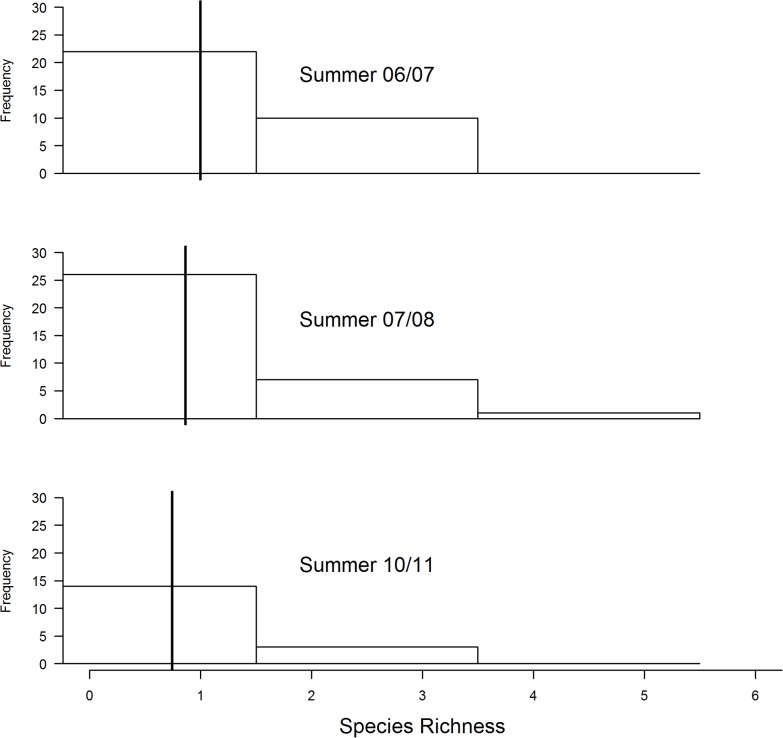
Comparison of bird species richness (birds that prefer heavy grazing only) at Ingula using data from summer surveys only. Year is treated as a fixed effect and transect as a random effect. **Bold** bars represent the estimated means.

**Fig 7:**
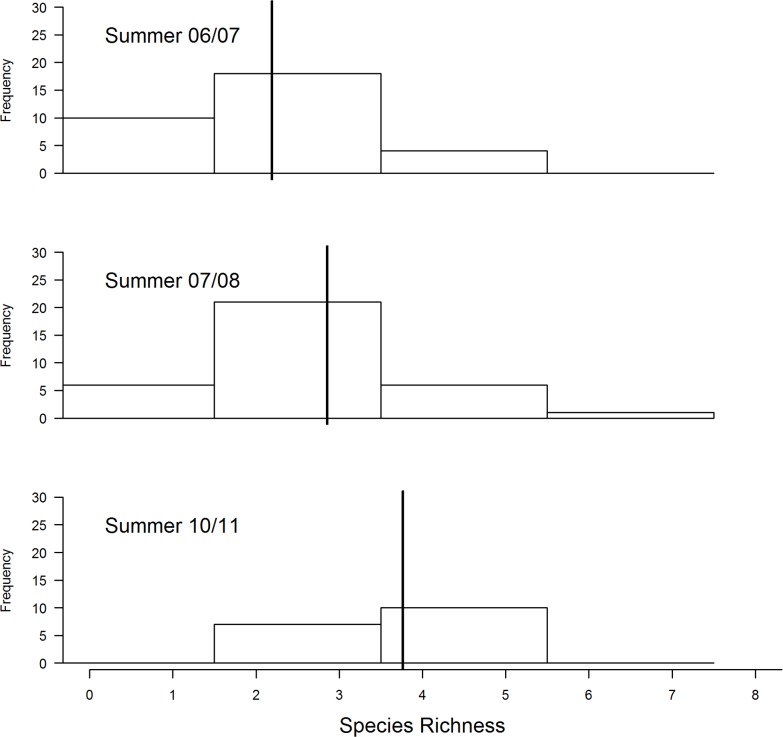
Comparison of bird species richness (birds that prefer moderate grazing only) at Ingula using data from three summer surveys. Year is treated as a fixed effect and transect as a random effect. **Bold** bars represent the estimated means.

When comparing bird species richness across the four seasons, summer had a highest number of species, followed by autumn and then winter while spring had the least species richness at Ingula (Fig 4). The model with season compared with a null model differed by a wide margin AICc = 166. Looking at the summer records alone and choosing only the species that depends on grassland to feed and breed, total bird species richness increased from 2006/07 through to 2007/08 and was highest during 2010/11 (Fig 5). For this group the model with year differed from the null model by a wide margin AICc = 091. When birds that prefers heavy grazing were analysed separately from birds that prefer Moderate grazing, the first group indicated a slightly decrease from to 2006/07–2010/11 (Fig 6) with an AICc = 4.4 comparing model with year and a null model. On the one hand birds that prefer moderate grazing showed the opposite trend with species richness highest in 2010/11 compared to 2006/07 (Fig 7). For this group the model with year differed significantly from the null model (AICc = 4).

**Fig 8:**
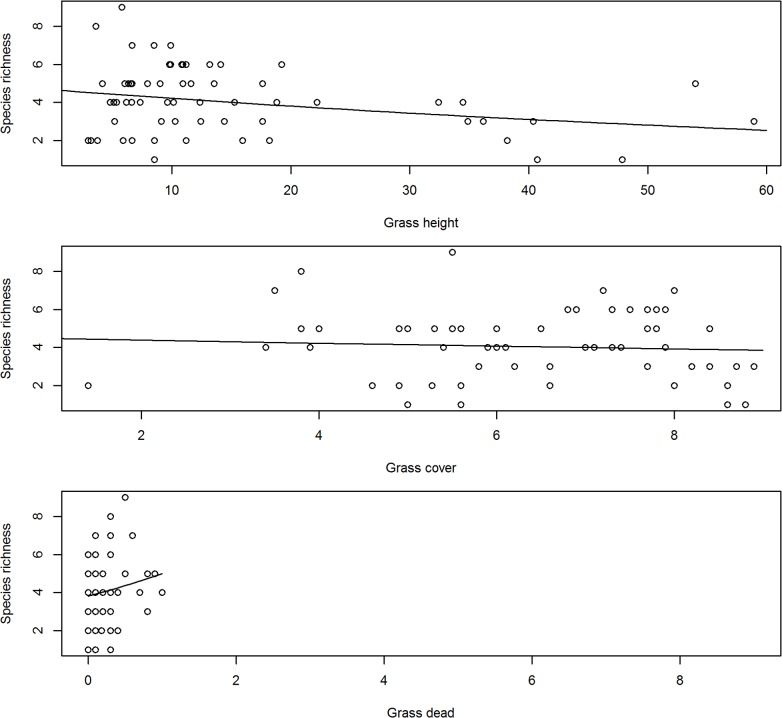
Response of birds species richness to grass height, cover and presence of dead grass along transect during the three summer surveys (2006/07, 2007/08 & 2010/11) at Ingula. The lines show the best fitting linear relationships (from Models ‘Cover’, ‘Height’ and ‘Dead’ in Table 3.

Grass height and cover are important grassland habitat variables frequently associated with bird’s habitat selection [46]. When analysing bird species richness in relation to the habitat (grass height, grass cover, dead grass, intensity of grazing or whether transect was burned or not) the model with grass height was selected as the best model explaining the changes in bird species richness irrespective whether we look at total bird species richness or treated birds that prefer heavy grazing separately (Tables 3 and 4). We found that a model with no covariates best explained species richness of birds that prefer moderate grazing (Table 5). We found little evidence relating bird species richness to grass cover or dead grass but some positive correlation with grass height (Fig 8).

**Table 3:** Model selection analysis relating total bird species richness in summer to transect habitat; constant model (Null), Fire (2 levels), Grazing (3 levels), Fire + Grazing, Grass cover, Grass height, Dead grass and Grass cover + Grass height. The models were generalised linear mixed models assuming a Poisson response and log link function. Transect was treated as a random effect in all models. K is the number of parameters in a model, Delta AIC is the differences in AICs.

Models	K	Log likelihood	Delta AIC	Akaike weight
Null	2	-125.455	1.605	0.163
Fire	3	-125.294	3.282	0.070
Grazing	4	-125.100	4.894	0.031
Fire + grazing	5	-124.887	6.470	0.014
Grass cover	3	-125.346	3.386	0.067
**Grass height**	**3**	**-123.653**	**0.000**	**0.364**
Dead grass	3	-124.845	2.385	0.110
Grass cover + Grass height	4	-123.355	1.404	0.180

**Table 4:** Model selection analysis relating bird species richness of birds that prefer heavy grazing in summer to habitat models; constant model (Null), Fire (2 levels), Grazing (3 levels), Fire + Grazing, Grass cover, Grass height, Dead grass and Grass cover + Grass height. The models were generalised linear mixed models assuming a Poisson response and log link function. Transect was treated as a random effect in all models. K is the number of parameters in a model, Delta AIC is the differences in AICs.

Models	K	Log likelihood	Delta AIC	Akaike weight
Null	2	-82.632	1.376	0.165
Fire	3	-82.474	3.060	0.071
Grazing	4	-81.266	2.644	0.088
Fire + grazing	5	-80.975	4.062	0.043
Grass cover	3	-82.525	3.162	0.068
**Grass height**	**3**	**-80.944**	**0.000**	**0.328**
Dead grass	3	-82.521	3.154	0.068
Grass cover + Grass height	4	-80.603	1.318	0.170

**Table 5:** Model selection analysis relating species richness of birds that prefer moderate grazing to habitat; constant model (Null), Fire (2 levels), Grazing (3 levels), Fire + Grazing, Grass cover, grass height, Dead grass and Grass cover + Grass height. The models were generalised linear mixed models assuming a Poisson response and log link function. Transect was treated as a random effect in all models. K is the number of parameters in a model, Delta AIC is the differences in AICs.

Models	K	Log likelihood	Delta AIC	Akaike weight
**Null**	**2**	**-101.6524762**	**0.000**	**0.311**
Fire	3	-101.5428757	1.781	0.128
Grazing	4	-101.593017	3.881	0.045
Fire + grazing	5	-101.5080766	5.711	0.018
Grass cover	3	-101.6270209	1.949	0.118
Grass height	3	-101.5166503	1.728	0.131
Dead grass	3	-101.0919824	0.879	0.201
Grass cover + Grass height	4	-101.5124918	3.720	0.048

## Discussion

We examined potential drivers of bird species richness at high-altitude grassland in eastern South Africa that underwent change in management. Our original intension to study the effects of fire on transects habitat and effects on bird species richness with controlled experiments was frustrated by lack of control over fire. Nevertheless we stuck to our protocol to systematically record vegetation and bird species occurrences within previously established random transects under the prevailing conditions.

In the absence of commercial farmers’ livestock, grass became higher leading to an increase in fuel load. Higher fuel loads apparently resulted in hotter fires when burning happened, which reduced grass cover on the ground [2]. Hot fires kill grass tufts resulting in patchiness. The observed increase in bare cover should be a concern for new management because it leads to an increased risk of erosion and invasion by woody plants [47]. Prescribed fire must be used cautiously with clear management objectives [48]. In this study our efforts to compare between burned and unburned control blocks as per [22] recommendations were frustrated by arson. If prescribed fire is not carefully implemented it could lead to decrease in grassland seed bank too [49] and therefore reduce grassland species diversity. Because burning happened under different weather conditions and different fuel loads in different years, it makes sense that the model with year and fire best explained differences the observed changes in grass cover and height.

With annual intense burning and relatively little grazing, we found the model with grass height alone as the best model explaining species richness both when total bird species richness is combined and also when birds that prefer heavy grazing were analysed separately from birds that prefer moderate grazing. The relatively high bird species richness in summer compared to other season agrees with previous findings [50] that high-altitude grasslands of eastern South Africa are predominantly used by birds in summer [50]. With no direct management intervention to increase bird species richness, the slight increase in bird species richness between 2005/06 and 2010/11 can only be related to stochastic phenomenon amongst years [51]. Same arguments goes for influence of grazing or burning or both on bird species richness because burning and grazing happened randomly with different intensities but this could partly be because there was not enough variation in these variables in our correlational study. Our results further suggest that individually, average grass height, grass cover and amount of dead grass were also not strongly correlated with species richness at Ingula (Fig 8), i.e. species richness does not clearly peak for any particular value of these habitat variables. However, one explanation for the lack of such a relationship could be that different species prefer different levels of grass cover and height. Based on our results we cautioned against associating birds species richness with grass height and cover directly this way. Instead, we suggest that habitat heterogeneity (mosaic of grass height and cover) is more important for species richness than average values [52–54]. Unfortunately, fire and grazing which determines habitat heterogeneity [55] were not under the control of management throughout this study and we could therefore not establish causal relationships between these management tools and habitat features. However, the increase in the amount of bare ground (Fig 1) in the absence of cattle was most likely due to an increase in hot fires under increased fuel loads [56]. The near absence of dead grass in summer (Fig 8), which is an important habitat feature for grassland birds to breed [5], can also be attributed to annual intense hot fires and should be a management concern too.

The diversity of species found in this study is high and includes a number of threatened species [4,14,45]. This study therefore supports the decision of designating the study area as an Important Bird & Biodiversity Area [4]. According to recent classification by Taylor et al. [45], several large threatened species have been added to the Ingula checklist (S1 Appendix), and are now feared declining and are subject of new monitoring programmes in South Africa [45,57,58]. For these species, new threats range from habitat loss to impact by powerlines amongst others [45].

### Habitat changes under new management

The observed decrease in summer grass cover (Fig 1) was unexpected because commercial livestock, which was thought to cause soil erosion and grassland degradation at Ingula [22], have been absent since the summer of 2005/06. In the absence of grazing, the observed increase in patchiness is likely related to hot fires that are fuelled by increased fuel load [22]. In addition, most fires at Ingula occurred during strong winds, which are likely to exacerbate the effects of hot fires on grasslands. Just as hot fires result in patchiness, heavy grazing leads to patchiness, which could lead to forb invasion [22]. Increasing bareness, together with lack of grazing, is likely to lead to dominance by fire-tolerant species, which would lead to reduced plant species diversity [59]. The few livestock that remained, which were owned by the tenants, did not result in heavy grazing. Although some transects might have not been burned at the time of the survey, in most cases fire would follow soon after. Mentis [22] recommended a fire return period of no less than two years.

### Factors that influence bird species richness at Ingula

Because Ingula is located within mid-altitude (1200 – 1700m asl) it is characterised by cold winters and wet summers. As a result, season alone has a profound effect on species richness/diversity, where most species use habitat in summer [50]. Based on our previous field observations we expected lowest avian species richness in spring too because we found the area to be almost deserted between winter to spring. Therefore, we did not expect increasing spring surveys would increase species richness at this time.

Grass height is important habitat covariate influencing total species richness (Table 3 and 4) while a null model was important for birds that prefer moderate grazing. This result agrees with our own field observations. Majority of birds that falls within moderate grazing category are small birds which do not get affected by changes in grass height as much as large birds do. For majority of birds visual inspection of habitat is more important than grass cover both in terms of avoidance of predation and also influence how birds makes use of habitat for breeding and foraging [60–62]. As a result grass height is more important than grass cover in terms of habitat selection and predation

Since the area hosts most species during summer, management should use fire and grazing so that habitat is suitable for birds to breed particularly in summer [46,55]. The study area consists of threatened birds that prefer heavy grazing and threatened birds that prefer moderate grazing indicating that management must maintain both types of habitats. Birds that benefit from heavy grazing and are threatened include; Southern Bald Ibis *Geronticus calvus* and Blue Cranes *Anthropoides paradiseus* both feed in fairly short grass early in the breeding season, while the latter species also uses short grass as a preferred habitat to breed. In the years when fire occurs late and in the absence of heavy grazing, we observed that, the small colony of Southern Bald Ibis at Ingula delays breeding while Blue Crane moves its nest into neighbouring farms where grass is mostly short but disturbance is high. Of the two small threatened birds Yellow-breasted Pipit *Anthus chloris* prefers moderate grazing while Rudd’s prefers heavy to moderate grazing. The last species is not included in (S1 Appendix) because it was seen outside transect survey period. The disappearance of Rudd’s Lark *Heteromirafra ruddi* from study site can possibly linked with little grazing after commercial livestock was withdrawn from the site. The area’s bird species richness include birds that breed outside summer [14], such as nationally Critically Endangered Wattled Crane *Grus carunculatus* which breeds from autumn to winter. This implies that management of these grasslands must also make habitat suitable outside summer. The study site can potentially harbour additional pairs of this bird [14], given the extent of habitat available for the species to breed.

Ingula management implemented relatively large fire breaks in early winter first to comply with Mentis [22] and secondly to contain the widespread burning. Even though ineffective at preventing run-away fires, these wide firebreaks, which were burned early, and other blocks remaining unburned until they were burned by arson late in the season, provided alternative refuges for birds so that birds did not leave Ingula altogether in the event of fire. However, as the entire property ended up burned, there was an absence of dry grass (Fig 8), of which majority of grassland birds need to construct nests timeously [5,12].

The alternative to estimating species richness is distance sampling. However, classical distance sampling methods do not estimate total species richness, i.e. the number of species that were never detected. We did explore distance sampling methods with our data but the methods need a minimum number of individuals detected and the analysis using distance sampling therefore provided us with much reduced number of species we could include. species richness is also a metric of many monitoring studies [24] and an objective of current Ingula grassland management.

## Management Implications & Conclusions

We suggest that management use fire and grazing to create a mosaic of burned and unburned blocks so that habitat become suitable for species of conflicting habitat needs to breed in summer within high-altitude grasslands. Frequent intense fires, heavy grazing or suppression fires all have undesirable effect on grassland habitat with negative impact on breeding birds too [63]. Our observational study should be complemented with experimental studies [64] to verify the cause effects of fire and grazing on bird species richness or habitat suitability fully.

The data analysis methods we used do not take into account the observation process, which can affect the results [65]. In particular, this study assumed constant detection probabilities across space and time. Although birds in general are relatively easy to find and identify, this is not the case with some of the grassland species, thus making it harder to ensure that detection probabilities remain constant. We believe that this problem was minimised in our case because all surveys were standardized and were conducted by one person who is familiar with birds of the study area choosing only days with favourable conditions. However, maintaining homogeneous detection probabilities when implementing these methods for long-term monitoring in our study area will be challenging given that future surveys will likely be carried out by different persons. The alternative to estimating species richness would be to concentrate on key grassland species and use distance sampling and relate the density of this species to habitat covariates [66]. And despite its shortcomings [67], species richness is a monitoring metric for many monitoring conservation projects. The current monitoring design could have been improved by visiting each transect at least twice per season [68]. However, owing to the large size of the study area, the unpredictable weather conditions and the fact that the census was done by one person, this was not possible for the present study.

## Supporting Information

S1 Appendix List of all species seen up to 150m from the transect line. (DOCX)
