# Supplementary material for: Drivers of Bird Species Richness within Moist High-Altitude Grasslands in Eastern South Africa
Source: PLoS One. 2016 Oct 5;11(10):e0162609. doi: 10.1371/journal.pone.0162609 (PMC5051898; doi:10.1371/journal.pone.0162609)
Supplement: S1 Appendix — (DOCX) [file pone.0162609.s001.docx]

**Appendix 1**: Grasslands birds seen within 150m during transect surveys between 2006 and 2011. Threatened species are in **bold**. Some bird species in the list do not qualify as typical grassland birds but were using grasslands at the time of survey. Some transects at the lower study site were close to bushes, while others have lone bushes that provided perches for birds that would otherwise not use grasslands. Regional Red List status is according to (45). Breeding status is only provided for the species for which evidence of breeding was confirmed and that they are grassland depended or they could be of management concern. Estimated time of breeding are indicated by Summer (S), Autumn (A), Winter (W) and Spring (Spr). Preferred habitat only provided for two types of birds **-** birds that prefer heavy grazing and those that prefer moderate grazing.

| **Species** | **Scientific name** | **National Red List status** | **Breeding status** | **Preferred habitat** |
| --- | --- | --- | --- | --- |
| Bishop, Southern Red | *Euplectes orix* | - | - | - |
| Bishop, Yellow-crowned | *Euplectes afer* | - | - | - |
| Bokmakierie | *Telophorus zeylonus* | - | - | - |
| Bulbul, Dark-capped | *Pycnonotus tricolor* | - | - | - |
| **Bustard, Denham's** | ***Neotis denhami*** | **Vulnerable** | Yes (S) | Heavy grazing |
| Buzzard, Jackal | *Buteo rufofuscus* | - | - | - |
| Buzzard, Steppe | *Buteo vulpinus* | - | - | - |
| Canary, Black-throated | *Crithagra atrogularis* | - | - | - |
| Canary, Cape | *Serinus canicollis* | - | - | - |
| Chat, Ant-eating | *Myrmecocichla formicivora* | - | Yes (S) | Heavy grazing |
| Chat, Sickled-winged | *Cercomela sinuata* |  | - | - |
| Cisticola, Cloud | *Cisticola textrix* | - | Yes (S) | Moderate grazing |
| Cisticola, Lazy | *Cisticola aberrans* | - | - | Moderate grazing |
| Cisticola, Levaillant's | *Cisticola tinniens* | - | - | - |
| Cisticola, Pale-crowned | *Cisticola aridulus* | - | - | - |
| Cisticola, Wing-snapping | *Cisticola ayrresii* | - | Yes (S) | Moderate grazing |
| Cisticola, Zitting | *Cisticola juncidis* | - | Yes (S) | Moderate grazing |
| **Crane, Blue** | ***Grus paradise*** | **Near Threatened** | Yes (S) | Heavy grazing |
| **Crane, Grey Crowned** | ***Balearica regulorum*** | **Endangered** | Yes (S) | - |
| **Crane, Wattled** | ***Grus carunculatus*** | **Critically Endangered** | Yes (A-W) | - |
| Crow, Cape | *Corvus capensis* | - | - | - |
| Crow, Pied | *Corvus albus* | - | - | - |
| Dove, Red-eyed | *Streptopelia semitorquata* | - | - | - |
| **Eagle, Martial** | ***Polemaetus bellicosus*** | **Endangered** | - | - |
| Egret, Cattle | *Bubulcus ibis* | - | - | - |
| Falcon, Amur | *Falco amurensis* | - | - | Heavy grazing |
| Fiscal, Common | *Lanius collaris* | - | - | - |
| Francolin, Red-winged | *Scleroptila africanus* | - | Yes (A-W) | Moderate grazing |
| Guineafowl, Helmeted | *Numida meleagris* | - | Yes (S) | Moderate grazing |
| **Harrier, Black** | ***Circus maurus*** | **Endangered** | Suspected (A -W) | - |
| Harrier, Montagu | *Circus macrourus* | - | - | - |
| Heron, Black-headed | *Ardea menalocepha* | - | - | - |
| Heron, Grey | *Ardea cinerea* | - | - | - |
| **Ibis, Southern Bald** | ***Geronticus calvus*** | **Vulnerable** | Yes (Spr - S) | Heavy grazing |
| Kestrel, Rock | *Falco rupicolus* | - | - | - |
| Kite, Black-shoudered | *Elanus caeruleus* | - | - | - |
| **Korhaan, White-bellied** | ***Eupodotus senegalensis*** | **Vulnerable** | Yes (S) | Heavy grazing |
| Lapwing, African Wattled | *Vannelus senegallus* | - | - | - |
| Lapwing, Blacksmith | *Vanellus armatus* | - | - | - |
| Lapwing, Black-winged | *Vanellus melanopterus* |  | - | Heavy grazing |
| Lapwing, Crowned | *Vanellus coronatus* | - | - | - |
| Lark, Eastern Long-billed | *Certhilauda semitorquata* | - | - | Heavy grazing |
| Lark, Pink-billed | *Spizocorys conirostris* | - | - | - |
| Lark, Red-capped | *Calandrella cinerea* | - | - | Heavy grazing |
| Lark, Rufous-naped | *Mirafra Africana* | - | - | Moderate grazing |
| **Marsh-Harrier, African** | ***Circus ranivorus*** | **Endangered** | Yes (S) | Wetland |
| Martin, Banded | *Riparia cincta* | - | - | - |
| Owl, African Marsh | *Asio capensis* | - | Yes (A-W) | Wetland |
| Pipit, African | *Anthus cinnamomeus* | - | - | Heavy Grazing |
| Pipit, Plain-backed | *Anthus leucophruys* | - | - | Heavy Grazing |
| **Pipit, Yellow-breasted** | ***Anthus chloris*** | **Vulnerable** | Yes (S) | Moderate grazing |
| Prinia, Drakensberg | *Prinia hypoxantha* |  | - | - |
| Quail, Common | *Coturnix coturnix* | - | Yes (S) | Moderate grazing |
| Quailfinch, African | *Ortygospiza attricollis* | - | Yes (A) | Moderate grazing |
| Quelea, Red-billed | *Quelea quelea* | - | - | - |
| Raven, White-necked | *Corvus albicollis* | - | - | - |
| Robin-Chat, Cape | *Cossypha caffra* | - | - | - |
| Rock-Thrush, Sentinel | *Montocola explorator* | - | - | - |
| Rush-Warbler, Little | *Bradypterus baboecala* | - | - | - |
| **Secretarybird** | ***Sagittarius serpentarius*** | **Vulnerable** | Yes (A-W) | Moderate grazing |
| Snipe, African | *Gallinago media* | - | - | - |
| Spurfowl, Swainson's | *Pternistis afer* | - | Yes (S) | Moderate grazing |
| Starling, Pied | *Spreo bicolor* | - | - | - |
| Starling, Red-winged | *Onychognathus nabouroup* | - | - | - |
| Stonechat, African | *Saxicola torquatus* | - | - | - |
| Stork, White | *Ciconia ciconia* | - | - | - |
| Sunbird, Malachite | *Nectarinia famosa* | - | - | - |
| **Vulture, Cape** | ***Gyps coprotheres*** | **Endangered** | - | - |
| Wagtail, Cape | *Motacilla capensis* | - | - | - |
| Waxbill, Common | *Estrilda astrild* | - | - | - |
| Wheatear, Mountain | *Oenanthe monticola* | - | - | - |
| Widowbird, Fan-tailed | *Euplectes axillaris* | - | - | - |
| Widowbird, Long-tailed | *Euplectes progne* | - | - | - |
| Widowbird, White-winged | *Euplectes albonotatus* | - | - | - |
